# The design and protocol of acupuncture for migraine prophylaxis: A multicenter randomized controlled trial

**DOI:** 10.1186/1745-6215-10-25

**Published:** 2009-04-24

**Authors:** Yan Zhang, Linpeng Wang, Huilin Liu, Nan Li, Jingdao Li, Jinghong Yi

**Affiliations:** 1 Beijing Traditional Chinese Medicine Hospital, Capital Medical University, Beijing, PR China; 2 Research Center of Clinical Epidemiology Affiliated to Peking University, Beijing, PR China

## Abstract

**Background:**

Many studies have already reported encouraging results in the prophylactic therapy of migraine by acupuncture, but there seems to be a lack of high quality randomized controlled trials from China. We design and perform a randomized controlled clinical trial to evaluate the efficacy of acupuncture compared with flunarizine in the prophylactic therapy of patients with migraine without aura in China.

**Methods:**

This trial is a multicenter, prospective, randomized controlled clinical trial. The 140 migraine patients are randomly allocated to two different groups. The acupuncture groups (n = 70) is treated with acupuncture and placebo medicine; while the control group (n = 70) is treated with sham acupuncture and medicine (Flunarizine). Both Flunarizine and placebo are taken 10 mg once per night for the first 2 weeks and then 5 mg once per night for the next 2 weeks. Patients in both groups receive 12 sessions of verum/sham acupuncture in 4 weeks.

**Discussion:**

The study design and the long term clinical practice of acupuncturists guarantee a high external validity for the results. The results of our trial will be helpful to supply the evidence on the efficacy of acupuncture for migraine prophylaxis in China.

**Trial Registration:**

The trial is registered at Controlled Clinical Trials: ISRCTN49839714.

## Background

Migraine leads to recurrent attacks of mostly unilateral, pulsating headache with associated symptoms such as photophobia, phonophobia, nausea, and vomiting. The diagnosis of migraine is based on criteria developed by the International Headache Society in 1988 and revised in 2004 – the International Classification of Headache Disorders II (ICHD II) [1]. Migraine prevalence are roughly 12% of the adults in western countries [2] and 4.2%–14.6% in China [3]. Epidemiological studies from several countries showed that the prevalence of migraine was 16–18% in women and 6–8% in men [4,5]. Approximately 90% of the migraine patients have moderate or severe pain, three quarters have a reduced ability to function during the headache attacks, and one-third require bed rest during their attacks [6].

Some patients with migraine are able to identify headache triggers. Triggers may be inconsistent or additive and are not specific to migraine. For example, menses is a trigger for 60% of female migraine patients. Stress or "let-down" after a stressful event, change in meal schedules or insomnia, and such environmental factors as loud noise, odors, or flickering lights may also precipitate migraine headache [7].

Migraine headache has a complex pathophysiology. Both vascular and neuronal mechanisms have been proposed. Although the mechanisms haven't been exactly known yet, several medications have been given to alleviate pain of patients. Pharmacotherapy can be abortive or prophylactic, and patients who experience frequent, severe headaches often require both approaches. Abortive medications used more than 2 days a week on a regular basis may lead to chronic daily or rebound headaches, so patients with headaches symptoms more than a few days a week should be on headache prophylactic therapy [7]. The goals of migraine prophylactic therapy are to (1) reduce attack frequency, severity, and duration, (2) improve responsiveness to treatment of acute attacks, and (3) improve function and reduce disability. Additional goals are to reduce costs and possibly prevent progression of episodic to chronic migraine. Prophylactic medication groups include β-adrenergic blockers, antidepressants, calcium-channel antagonists, serotonin antagonists, anticonvulsants, nonsteroidal anti-inflammatory drugs, and others (vitamins, minerals, natural products) [8]. Calcium-channel antagonists such as flunarizine have been used commonly in China. Choice is based on efficacy, adverse events, and coexistent conditions. However, most of medications have side effects and contraindication that may limit their use.

Acupuncture, which is one of the main treatment modalities of Traditional Chinese Medicine (TCM), has been used for both the prevention and treatment of diseases for over three thousand years [9]. Although how acupuncture works is still unknown [10], the range of indications of acupuncture (especially for pain management), the variety of different acupuncture methods and the probability that the effects are made up of a specific and a nonspecific component have been acknowledged by more and more western countries [11].

Many studies have already reported encouraging results in the therapy of migraine by acupuncture. In 2001 a systematic review on acupuncture for idiopathic headache had underlined the existing evidence that acupuncture had an effective role in the treatment of patients with migraine [12], but methodological or reporting shortcomings were found in the majority of the studies, so the evidence was insufficient to determine the efficacy of acupuncture compared to medication, or to wait list control or sham acupuncture, in the management of patients with migraine. Since that systematic review, several large, randomized trials on the effectiveness of acupuncture as treatment for migraine prophylaxis have been published in western countries, but there seems to be a lack of high quality randomized controlled trials from China. China is the origin of acupuncture and the country where acupuncture has been used most commonly in clinical practice. Therefore, we are performing a randomized controlled trial to investigate the efficacy of acupuncture compared with flunarizine in patients who have frequent migraine attacks in China.

## Methods

This trial is a multicenter prospective, randomized, controlled clinical trial. The central randomization is performed by Research Center of Clinical Epidemiology Affiliated to Peking University in China, which uses block randomization to generate the random allocation sequence and prepares a predetermined computer-made randomization opaque sealed envelopes. The opaque sealed envelopes are numbered consecutively and were connected into a strain. It is requested that each envelope should be separated from the strain and then be opened in sequence only after baseline period when the patient has been registered in the trial. Patient and assessors are blinded (blinded telephone interviewers) with regard to the acupuncture treatment administered. The trial is executed in the following five hospitals from June 2007 to June 2009: Beijing Traditional Chinese Medicine Hospital Affiliated to Capital Medical University, Peking University Third Hospital, Beijing Tiantan Hospital Affiliated to Capital Medical University, Huguosi Hospital Affiliated to the Beijing University of Chinese Medicine and Dongzhimen Hospital Affiliated to the Beijing University of Chinese Medicine. Physicians who enroll participants and assessors who collect data in these five hospitals must have a 3-day training seminar concerning treatment modalities and trial's documentation prior to the trial to make sure all practices at each of the five hospitals were the same. Periodic check-up contained the coincidence of the practices taken in every hospital.

This trial is performed according to the principles of the Declaration of Helsinki (Version Edinburgh 2000). The trial protocol has been approved by the Research Ethical Committee of Beijing Traditional Chinese Medicine Hospital Affiliated to Capital Medical University on May 2007.

### Participants

One hundred and forty patients with migraine without aura enrolled in the trial are recruited from outpatient in acupuncture clinics of the five hospitals. The main inclusion criteria are: patients suffering from migraine without aura (more than 2 migraine attacks in 4 weeks), diagnosed according to diagnostic criteria of the International Headache Society [1]; male or female; aged 18–65 years; patients who had not used acupuncture or drugs with migraine prophylactic effects within the last 3 months; patients who have given written informed consent.

The main exclusion criteria are: tension-type headache, cluster headache and other primary headaches; secondary headache and other neurological diseases; neuralgia of the face or head; pregnancy, nursing mother or insufficient contraception; use of prophylactic migraine medication in the last 3 months; therapy with β-blocker in the last 3 months; intake of antipsychotic or antidepressant drugs; participation in another clinical trial; having family history of depression, Parkinson disease and other extra-pyramidal diseases.

According to the previous studies in China and our pilot study, we anticipate that the proportion of responders [13] (50% or greater reduction in attack frequency) would be 65% [14,15] in control group and 90% [16] in acupuncture group. Based on 0.9 power to detect a significant difference (α = 0.05, two-sided), 56 patients are required for each group calculated by EpiCalc 2000 Version 1.02. To compensate for dropout patients, we plan to enroll 70 patients per group.

### Procedures

**Figure 1:**
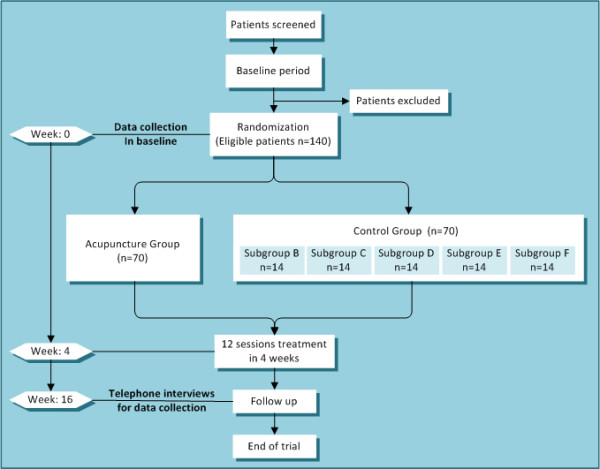
**Trial profile**.

According to predetermined randomization envelopes, 140 eligible patients are randomly allocated to acupuncture group or control group (see Figure 1). The patients have an equal probability of being assigned to either of two groups. Patients need to record headaches and usage of acute medication in headache diary from baseline period to the 16^th ^week after randomization. According to Case Report Form including all outcome measures, endpoints are collected separately 16 weeks after randomization by blinded telephone interviewers in the five hospitals to evaluate the long term effect of acupuncture prophylaxis.

### Intervention

In addition to rigorous methodological quality, both verum acupuncture and sham acupuncture interventions have been proposed based on a broad literature review and a consensus with acupuncture experts in Beijing Traditional Chinese Medicine Hospital. We conducted a pilot study with sample size 32 per group to confirm the efficacy of verum acupuncture compared with sham acupuncture. Besides, the interventions of two groups are based on the experiences of physicians who had worked with acupuncture for 20 years.

In the acupuncture group, 3 acupuncture sessions per week are carried out and placebo medicine is taken once a night for 4 weeks. In the control group, medicine (flunarizine) once a night for 4 weeks is given and 3 sham-acupuncture sessions are given per week. There are several common approaches of verum/sham acupuncture in both groups including: bilateral points; usage of disposable, sterile steel needles (0.32 mm × 40 mm); 10–12 needles used each time; skin disinfection with 75% alcohol; needles retention for 30 minutes and no moxibustion or electrical stimulation. Patients of both groups received 12 sessions of verum/sham acupuncture in 4 weeks.

The placebo medicines have exactly the same appearance as true medicines (flunarizine). Both Flunarizine and placebo are taken 10 mg once per night for the first 2 weeks and then 5 mg once per night for the next 2 weeks. All patients are allowed to treat acute headaches as need, but the type and dose of acute medications should be recorded in a headache diary.

#### Verum Acupuncture

**Figure 2:**
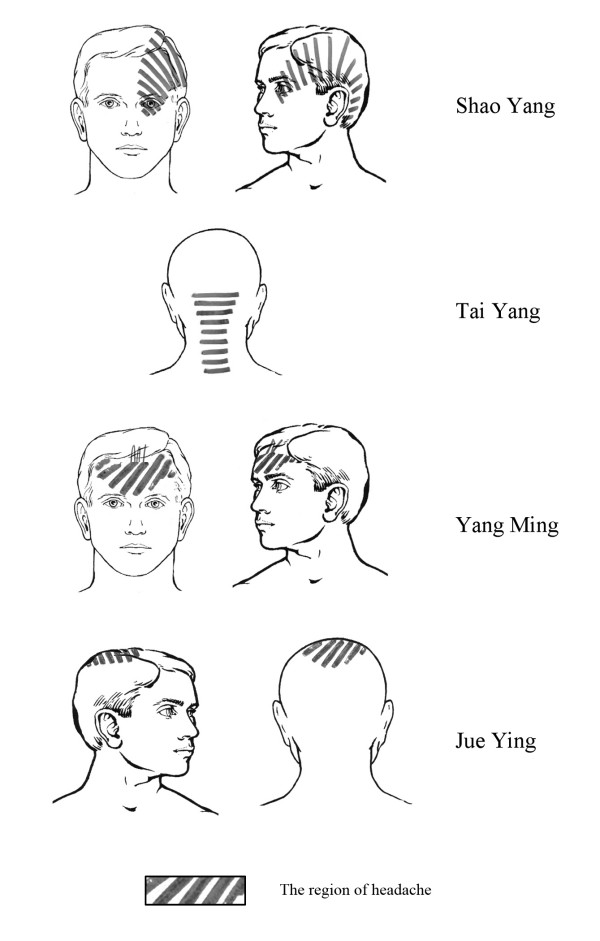
**Syndrome differentiation of meridians in headache region**.

The obligatory points used in acupuncture group include DU20 (Baihui), DU24 (Shenting), GB13 (Benshen), GB8 (Shuaigu), SJ20 (Jiaosun) and GB20 (Fengchi). Additional points can be chosen according to syndrome differentiation of meridians (see Figure 2):

1. Shaoyang headache (TE-GB): SJ5 (Waiguan), GB34 (Yanglingquan).

2. Yangming headache (LI-ST): LI 4 (Hegu), ST 44 (Neiting).

3. Taiyang headache (SI-BL): BL 60 (Kunlun), SI 3 (Houxi).

4. Jueyin headache (PC-LR): LR3 (Taichong), GB40 (Qiuxu).

5. Nausea and vomiting: PC6 (Neiguan).

6. Dysphoria and susceptibility to rage: LR3 (Taichong).

Needles are inserted 10–15 mm in depth and manually manipulated by rotation methods to produce a characteristic sensation known as "De Qi".

#### Sham Acupuncture

To make the quantity of stimulus uniform between two groups, the decision is made to use the same kind, size and number of needles for sham acupuncture as which are used in verum acupuncture. However, the sham points selected would be confirmed as the distant non-meridian points that are defined as 3 mm apart from the points without effects on headache. The procedure for developing the sham acupuncture protocol contains three steps:

1. Literature search: Based on the search and analyses of 26 Chinese acupuncture ancient articles, 3 Chinese acupuncture textbooks and more than one hundred acupuncture modern articles, the points with no reference to effects on headaches have been enumerated.

2. Point selection: To avoid the effects on headaches and the effects of alleviating pain, the points in the head, trunk, hands and feet are excluded. And then 30 points without effects on headaches are extracted and the locations 3 mm apart from these points are defined as sham points, which are used in the control group.

3. Sham points subgroup: 30 sham points are randomly assigned to five subgroups of control groups which are labeled from B to F and recorded in the predetermined computer-made randomization sealed envelope. Each subgroup has two points on arms and three points on legs. The patient who belongs to the control group will be assigned to one of subgroups and the five points in this subgroup will be used on this patient in the whole treatment period. The details of the sham points in the control group are in Table 1.

**Table 1:** The sham points in the control group.

Subgroup	Code	Arm		Leg		
1	B	LI15†	LI14‡	KI9†	GB33†	LR7†
2	C	SJ6‡	SJ8†	SP7†	KI10†	GB38†
3	D	LI13‡	PC5‡	GB35†	GB37†	GB39†
4	E	LU5‡	PC2‡	SP9†	KI7†	LR6†
5	F	LI12‡	HT4‡	ST32‡	ST38‡	ST39‡

† Backward 3 mm from the point; ‡ Outward 3 mm from the point.

### Outcome measures

The efficacy of acupuncture for migraine prophylaxis is assessed by the following primary outcome measures:

1. Change in frequency and duration of migraine attacks [13].

2. The proportion of responders, defined as the proportion of patients with a reduction of migraine days by at least 50% [13].

3. Visual Analogue Scale (VAS) to assess the severity of migraine pain [17].

4. Short-Form of McGill Pain Questionnaire (SF-MPQ) [17,18].

The secondary outcome measures include:

1. The summary scales of the SF-36 [19,20].

2. Intake of acute-medication [13].

3. Number of patients with adverse side effects [21].

4. Change in the frequency of nausea and vomiting [22].

The outcome measures above will be assessed before the treatment, the week of the last acupuncture session and 12 weeks after the last acupuncture session.

### Statistical analysis

All analyses will be done on the intention-to-treat group of participants who have been randomized regardless of whether they receive any treatment. Missing data will be replaced according to the principle of the last observation carried forward. A variance analysis reject the global hypothesis H_0_: "There is no difference in the success probability between two groups." The significant level used for the statistical analysis is 5%, therefore P < 0.05 indicates significance. Analysis of variance (ANOVA) for repeated measures is used to compare the two groups. Comparisons within groups for the outcomes in each one of the time point were done using the Tukey's post hoc test of Multivariate Analysis of Variance (MANOVA). In the case of proportions, a *chi-square test *is applied. Stratified analysis within five different centers will be performed to control confounding factor if necessary. All analyses are performed using the Statistical Package for the Social Sciences (SPSS) software program (version 13.0) for Windows XP.

## Discussion

The question we put forward in this clinical trial is: "Is acupuncture prophylaxis as effective as flunarizine for migraine?" Flunarizine is a drug classified as a calcium channel blocker, which is commonly used in China for migraine prophylaxis. A large sample size study considered that flunarizine is safe and effective for the prophylactic treatment of migraine in China [23]. Therefore, we compare the efficacy of acupuncture with flunarizine which is chosen as a standard drug therapy. The strength of our trial is strictly concealed randomization, the successful blinding of the patients, data collection in diaries. Moreover, in this trial all physicians applying acupuncture treatments have more than 20 years of clinical experience of acupuncture.

According to the theory of traditional Chinese medicine, migraine headaches can be considered as syndromes of meridians [24]. So the acupuncture intervention is given based on the concept of syndrome differentiation of meridians. As an important part of the theory of meridians, syndrome differentiation of meridians has been performed in clinical practice for thousands of years. The aim of syndrome differentiation of meridians is to analyze the involvement of the meridians according to the distribution and indication of meridians for the purpose of providing evidence for selecting the needling methods [25]. For example, most migraine patients with headache on the temporal side, where the distribution of meridians of foot-Shaoyang, should pertain to Shaoyang headache, while some migraine patients with headache on the forehead should pertain to Yangming headache, because forehead is the distribution of meridians of foot-Yangming. In the case of Shaoyang headache, SJ5 and GB34 can be added besides obligatory points that have to be needled in the trial.

The protocol of sham points is developed in our trial according to international considerations about the methodological problems of sham acupuncture [26]. To avoid any effects on headache, firstly we searched points without effect on headache from both ancient and modern literature. This method of selecting sham points can be seen in Alecrim (2008) [22], but our research must be more extensive, because we searched many Chinese articles, particularly ancient books, which have not yet been translated into English. Secondly, as Linde (2005) [27] did, distant non-meridian point is used as sham point by needling 3 mm apart from the points without effect on headache to further avoid the meridian affects in the control group. Last but not least, there are 30 points without effects on headache extracted, but only 5 points will be used to each patient in control group. Points, which are chosen and made up as a control subgroup, are determined by computer-made randomization. This process of confirming sham points could further eliminate potential effects on headache.

To mask the patients in our trial, sham acupuncture is adopted in the control group while placebo medicine is used in acupuncture group, which is considered to benefit the homogeneity of potential psychological effects and improve compliance of patients in China. Alecrim (2008) [22], Diener (2006) [13] and Linde (2005) [27] reported that sham acupuncture had similar effects to verum acupuncture in reducing migraine headaches and both interventions were effective. They thought that several nonspecific factors could explain the high improvement of the sham group, such as the patient's belief in positive results of the acupuncture treatment and the frequent patient's contact with the acupuncturist. Even though in our trial these factors have been standardized as strictly as possible and the selection of sham points is different from previously published trials, we cannot rule out that sham acupuncture intervention may have some physiological effects. The nonspecific physiological effects of needling may include local alteration in circulation and immune function as well as neurophysiological and neurochemical responses as reported [28,29]. Moreover, the placebo effects may be higher in pain sufferers than in patients suffering from other complaints [30] and invasive technical proceedings may have higher analgesic effects than oral drugs [31,32]. Our design therefore might prefer the flunarizine group.

Acupuncture has been used for alleviating acute/chronic pain for thousands of years. The efficacy of acupuncture as abortive therapy for patients with migraine headaches has been proved by many studies [12]. Some studies suggested that sumatriptan was more effective than acupuncture at relieving headache [33]. Therefore, the purpose of this trial is not to discuss the efficacy of acupuncture as painkiller but evaluate the efficacy on preventing migraine attack. The results of our trial will be helpful to supply the evidence on the efficacy of acupuncture for migraine prophylaxis in China.

## Competing interests

The authors declare that they have no competing interests.

## Authors' contributions

LW, YZ, HL participated in the design of the trial, in plans for the analysis of the data, and in drafting the manuscript. NL, JL, JY participated in the design of the trial. All authors read and approved the final manuscript.
